# Lunatic Asylum on an Improved Plan: The Midland Retreat

**Published:** 1836-01

**Authors:** 


### LUNATIC ASYLUM ON AN IMPROVED PLAN—THE MIDLAND RETREAT.

WE have received a Prospectus of an intended Establishment for Lunatics thus designated, which, if carried into effect on the principles and plan announced, cannot fail to be equally advantageous to the profession and to the public. The two introductory paragraphs, which we extract, explain briefly the nature of the proposed institution, and the evils it is intended to remedy.

"It has long been felt throughout the profession, that there is nothing which involves medical men in circumstances so unsatisfactory as the occurrence of insanity in any of their private patients. Without detailing all these circumstances, it is only necessary to observe that it almost always becomes expedient to remove the patient from home, and to abandon the care of him to strangers; often to those residing at a distance, reluctant to communicate with the practitioner who has previously attended the patient, and knows his habits and peculiarities. Although many well-conducted establishments exist in this country, it yet sometimes happens that patients are hastily consigned to houses in which medical treatment is disregarded, and the chief object seems to be to derive a permanent income from the patient's continued confinement; and, in all cases, the patient is taken away from his family medical attendant.

"To obviate, as much as possible, this entire separation of the practitioner from his patient; to secure the remuneration for superintending the treatment of each patient exclusively to medical men; and at the same time to afford an unexceptionable Retreat, from whence regular reports of each patient will be transmitted to his or her former medical attendant; an institution, in short, the entire property and management of which shall be vested in medical men, constituting an extensive partnership or joint-stock company; it is proposed that such a partnership or company be formed, for the institution of such an establishment in the centre of England, to be called the MIDLAND RETREAT."

